# Dex modulates the balance of water-electrolyte metabolism by depressing the expression of AVP in PVN

**DOI:** 10.3389/fphar.2022.919032

**Published:** 2022-08-23

**Authors:** Wenzhi Yang, Hao Li, Zhongle Cheng, You Lu, Wuli Li, Jun Feng, Liecheng Wang, Juan Cheng

**Affiliations:** ^1^ Department of Physiology, School of Basic Medical Sciences, Anhui Medical University, Hefei, China; ^2^ Shenzhen-Hong Kong Institute of Brain Science-Shenzhen Fundamental Research Institutions, Shenzhen Neher Neural Plasticity Laboratory, Shenzhen Key Laboratory of Drug Addiction, the Brain Cognition and Brain Disease Institute, Shenzhen Institute of Advanced Technology, Chinese Academy of Sciences (CAS), Shenzhen, China; ^3^ Department of Clinical Laboratory, The First Affiliated Hospital of Anhui Medical University, Hefei, China; ^4^ College and Hospital of Stomatology, Anhui Medical University, Hefei, China

**Keywords:** dexmedetomidine, paraventricular nucleus, arginine vasopressin, electrolytes level, thirsty, renal function, urine, plasma osmolality

## Abstract

Dexmedetomidine (Dex) is a highly selective α2 adrenergic agonist used in clinical anesthesia. Studies have shown that Dex can act on the collecting duct and reduce the body’s water reabsorption, thereby increasing water discharge. However, the specific mechanism of Dex on water homeostasis remains unclear. The hypothalamus is the regulatory center of water and salt balance and secretes related neurochemical hormones, such as arginine vasopressin (AVP), to regulate the discharge of water and salt. The paraventricular nucleus (PVN) and supraoptic nucleus (SON) in the hypothalamus are also considered to be the key targets of the thirst loop. They are responsible for the secretion of AVP. The suprachiasmatic nucleus (SCN) is also one of the brain regions where AVP neurons are densely distributed in the hypothalamus. This study used C57BL/6J mice for behavior, immunofluorescence, and blood analysis experiments. Our results showed that Dex could not only depress the expression of AVP in the PVN but also reduce serum AVP concentration. The animal water intake was decreased without impairing the difference in food consumption and the urine excretion was enhanced after the intraperitoneal injection of Dex, while AVP supplementation restored the water intake and inhibited the urine excretion of mice in the Dex group. In addition, the renin-angiotensin-aldosterone system is vital to maintaining serum sodium concentration and extracellular volume. We found that serum sodium, serum chloride, serum aldosterone (ALD) concentration, and plasma osmolality were decreased in the Dex group, which inhibited water reabsorption, and the plasma osmolarity of mice in the Dex group supplemented with AVP was significantly higher than that in Dex group. We also found that Dex significantly increased the concentration of blood urea nitrogen and decreased the concentration of creatinine within the normal range of clinical indicators, indicating that there was no substantive lesion in the renal parenchyma. These results showed that Dex could modulate the balance of water-electrolyte metabolism by depressing the expression of AVP in PVN without impairing renal function.

## 1 Introduction

Dexmedetomidine (Dex) is a potent and highly selective α2 adrenergic agonist with sedative, analgesic, and anti-anxiety functions that are widely used in clinical anesthesia (Weerink et al., 2017). It can also be used to eliminate delirium, adjust sleep structure, and reduce sympathetic excitement and inflammatory responses (Bindra et al., 2019; Gasim et al., 2021; Tasbihgou et al., 2021; Xue et al., 2021). Dex can inhibit osmotic water permeability in the rat cortical collecting duct, thus increasing body water discharge (Rouch et al., 1997). Water-salt balance is crucial to the steady state of the internal environment (Rao et al., 2019). However, whether Dex can modulate the intake or discharge of water and salt to regulate water-electrolyte metabolism is still unclear.

Water homeostasis is a key process, involving the coordinated action of the hypothalamus, including thirst mechanism, control of arginine vasopressin (AVP) release, and kidney where water is reabsorbed or excreted in urine (Christ-Crain et al., 2021). Hypothalamus, as the regulating center of water and salt balance in the body, is also the thirst center, which balances the water between the control of water intake behavior and loss from kidney or non-kidney in the body (Cakir and Nillni, 2019; Verbalis, 2020). When osmolality increases, the synthesis of AVP increases in magnocellular and parvocellular neurons, whereas the magnocellular neurons are mainly located in the paraventricular nucleus (PVN) and supraoptic nucleus (SON) (Leng et al., 1999; Treschan and Peters, 2006). The axons of magnocellular neurons release AVP into the circulation of the posterior pituitary lobe, and the axons of parvocellular neurons release AVP into the pituitary portal vein circulation (Scott and Dinan, 1998; Leng et al., 1999; Treschan and Peters, 2006). AVP can promote the reabsorption of water and reduce the excretion of sodium and contract blood vessels (Sladek, 2004; Henderson and Byron, 2007; Armstrong and Johnson, 2018). In the peripheral circulatory system, two types of vasopressin receptors, vasopressin 1 receptors (V1Rs) and vasopressin 2 receptors (V2Rs), are expressed in vascular smooth muscle cells in the heart and kidney. In particular, the V2Rs in the kidney functionally regulate water metabolism and further play a crucial role in the initiation and progression of renal disease (Barberis et al., 1998; Birnbaumer, 2000; Bolignano and Zoccali, 2010).

In terms of thirst, the subfornical organ (SFO) is the key brain region that mediates thirst response. When mice are dehydrated, SFO neurons are activated. Since unknown signals from the mouth may act through the trigeminal ganglion, drinking water will almost immediately inhibit this activity (Bankir et al., 2017). SFO neurons project to other median preoptic areas (MnPO) and Organum vasculosum laminae terminalis (OVLT) nucleus and then transmit to vasopressin neurons in the downstream PVN and SON to control the expression and release of AVP (Oka et al., 2015; Bankir et al., 2017). The expected signals of thirst and AVP release converge on the same steady-state neurons, especially the neurons of the SFO (Bankir et al., 2017). When the blood osmolality rises, the neurons in SFO are activated to produce thirst, which will also induce the increase in AVP release. In other words, the production process of thirst and AVP is often synergistic (Augustine et al., 2018). The hypotonic state of blood is also considered to be an inhibitor of thirst (Gizowski and Bourque, 2018).

AVP is very important for the regulation of water-salt balance as above, the AVP neurons is mainly located in the three brain regions of PVN, SON, and SCN (Verney, 1947; Silverman and Zimmerman, 1983; Ono et al., 2021). In mammals, Dex mainly plays a major role via the α2-adrenoceptor (Weerink et al., 2017), and these receptors are also densely distributed in the PVN, SON, and SCN (Unnerstall et al., 1984). So whether Dex can regulate the intake and discharge of water and salt as well as the expression of AVP, and via what kind of hormone to regulate have not yet been clarified. In this study, three different doses of Dex (50 μg/kg, 100 μg/kg and 200 μg/kg) were used through intraperitoneal injection (Zhang et al., 2015). Our results showed that Dex significantly decreased water intake and promoted urine excretion in both dose-dependent pharmacologic and physiological contexts, while the inhibitory effect of water intake and the urination promoting effect will be significantly mitigated after AVP supplementation. We found that Dex significantly decreased the expression of AVP in the PVN. Besides, Dex also significantly reduced AVP concentration in serum. Moreover, our study found that Dex decreased aldosterone (ALD), sodium, chloride concentrations in serum and plasma osmolality, which infers Dex can increase the discharge of water and salt directly or indirectly. All of our findings indicate that Dex could modulate the balance of water-electrolyte metabolism by depressing the expression of AVP in PVN without impairing renal function.

## 2 Results

### 2.1 Dex inhibited the activities of AVP neurons in the PVN

**FIGURE 1:**
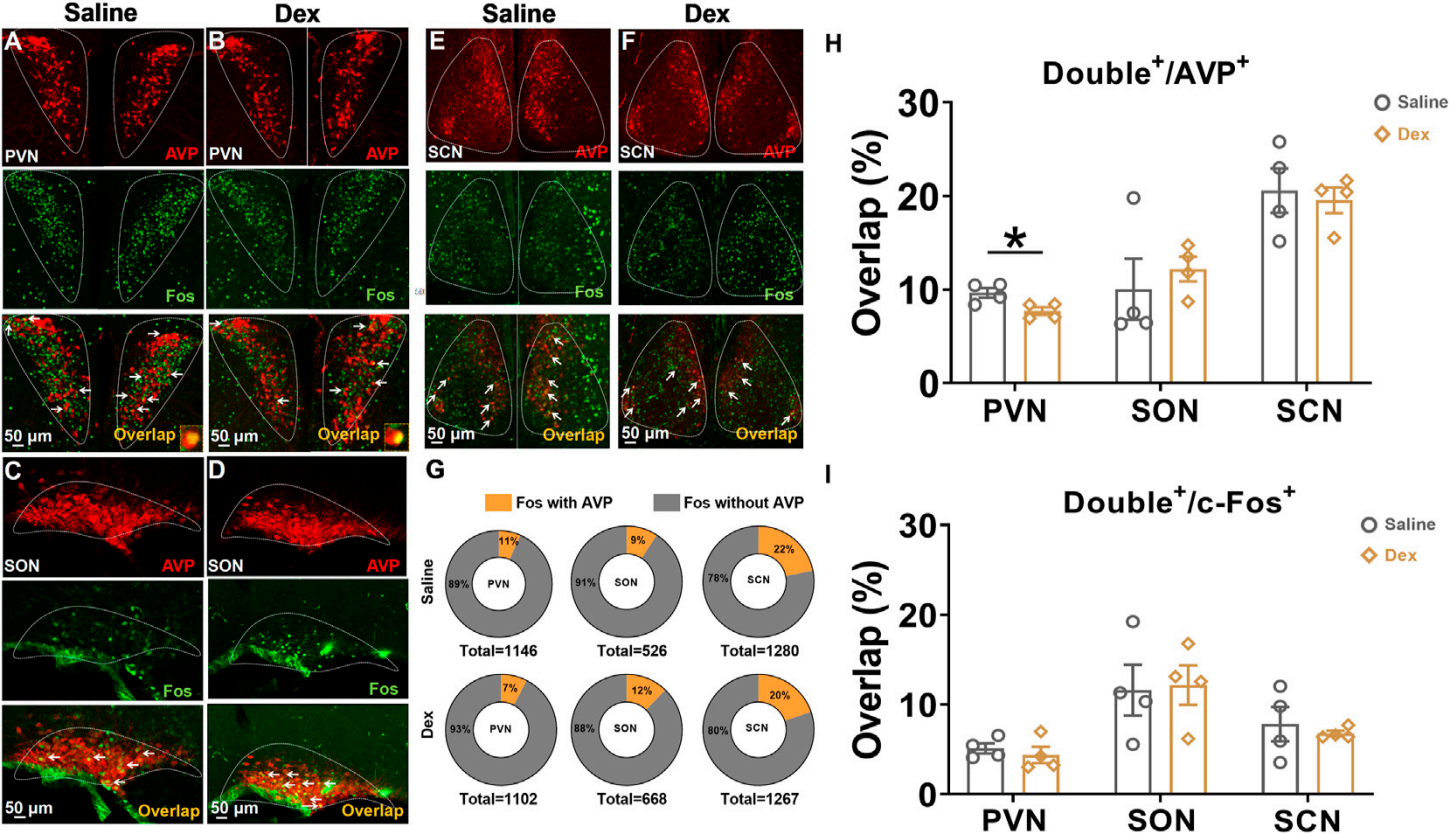
The AVP neurons in the PVN were depressed by Dex **(A–F)**. Representative double staining photomicrographs of c-Fos expression (green) and AVP expression (red) (scale bar, 50 µm) in SCN, PVN and SON treated saline or Dex (100 μg/kg). **(G)** The illustration shows the number of c-Fos colabeled with or without AVP neurons in the SCN, PVN and SON. **(H)** The proportion of double-labeled neurons to AVP neurons treated with saline or Dex in the SCN, PVN and SON. **(I)** The proportion of double-labeled neurons to c-Fos treated with saline or Dex in the SCN, PVN and SON. The values are presented as the mean ± SEM (*n* = 4, each group), *p < 0.05 indicates a significant difference between the saline and Dex treatment groups assessed using the unpaired two-tailed Students *t* test.

AVP and its receptor are associated with body water homeostasis. Therefore, we performed costaining of AVP neurons in the SON, PVN, and SCN located in the hypothalamus. The results showed that in the PVN, the proportion of double-labeled neurons to c-Fos in the Dex group was not significantly different from that in the saline group (*n* = 4, *p* > 0.05) (Figures 1A,B,I); however, the proportion of colabeled AVP and c-Fos in the Dex group was significantly decreased compared with that in the saline group (*n* = 4, *p < 0.05) (Figures 1A,B,H). However, no difference was found in the SON or SCN between the two groups (*n* = 4, *p* > 0.05) (Figures 1C–F,H,I). In addition, it can be seen that the total number of c-Fos or AVP neurons in PVN, SON, and SCN was almost the same in the saline group and Dex group (Figure G). The above results illustrated that Dex significantly suppressed AVP expression in the PVN.

### 2.2 Dex decreased plasma AVP concentration and dose-dependently decreased water intake

**FIGURE 2:**
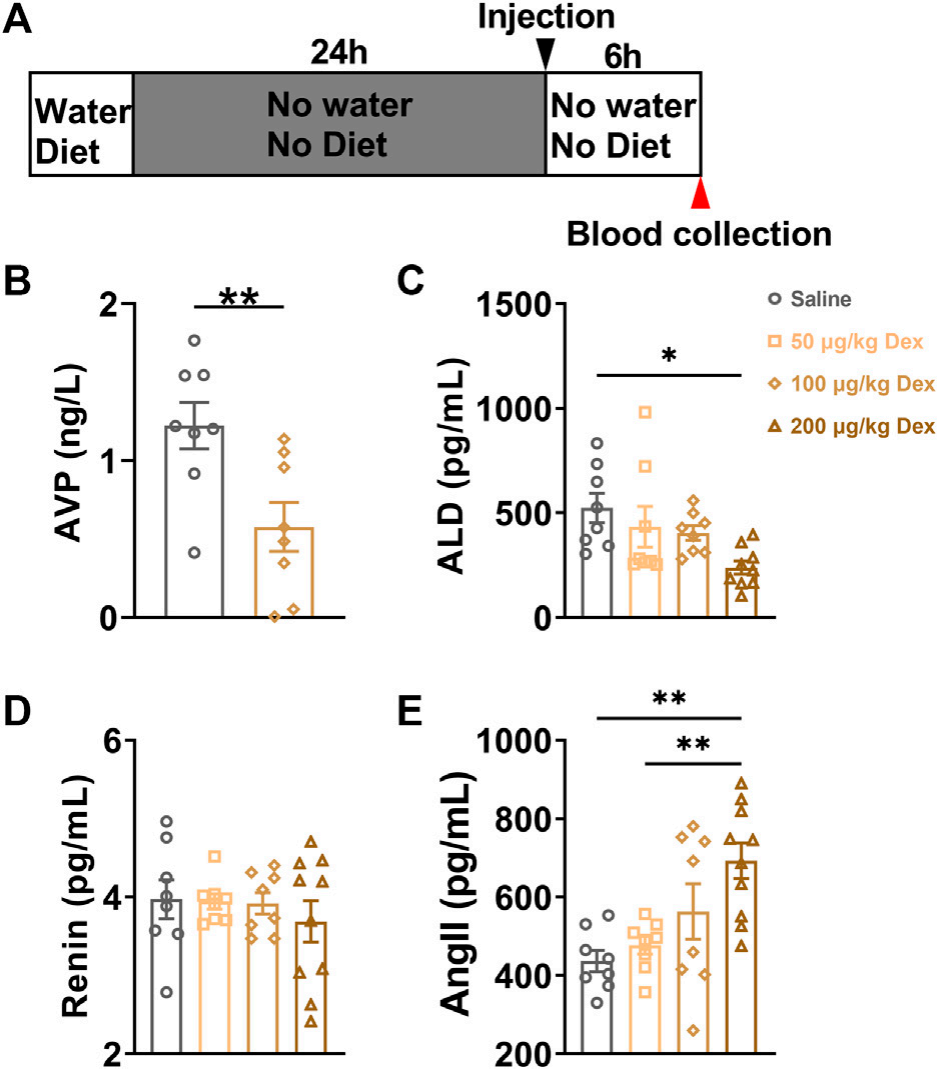
Dex decreases the serum AVP and ALD concentration in peripheral blood. **(A)** The experimental protocol for blood sample collection after saline or Dex administration. **(B)** Serum AVP concentration in the saline and Dex groups (100 μg/kg). **(C–E)** Levels of hormones in the renin-angiotensin-aldosterone system after i. p. Saline, 50 μg/kg Dex, 100 μg/kg Dex and 200 μg/kg Dex. The values are presented as the mean ± SEM (*n* = 8 in the saline, 50 μg/kg Dex and 100 μg/kg Dex groups, *n* = 10 in the 200 μg/kg Dex group), **p < 0.01 and ***p < 0.001 indicate significant differences among the saline and Dex groups assessed using unpaired two-tailed Student’s *t* test in Figure 2B, one-way ANOVA in Figure 2 **(C–E)**.

Based on the calculation result of colabeled AVP neurons in the hypothalamus after Dex injection, we conducted the plasma AVP concentration of blood samples detected with the ELISA experiment. The blood of animals in each group was collected at the 7th hour after saline or Dex (100 μg/kg) administration (Figure 2A). The results showed that Dex significantly decreased the plasma AVP concentration in peripheral blood (*n* = 8, **p < 0.01) (Figure 2B).

**FIGURE 3:**
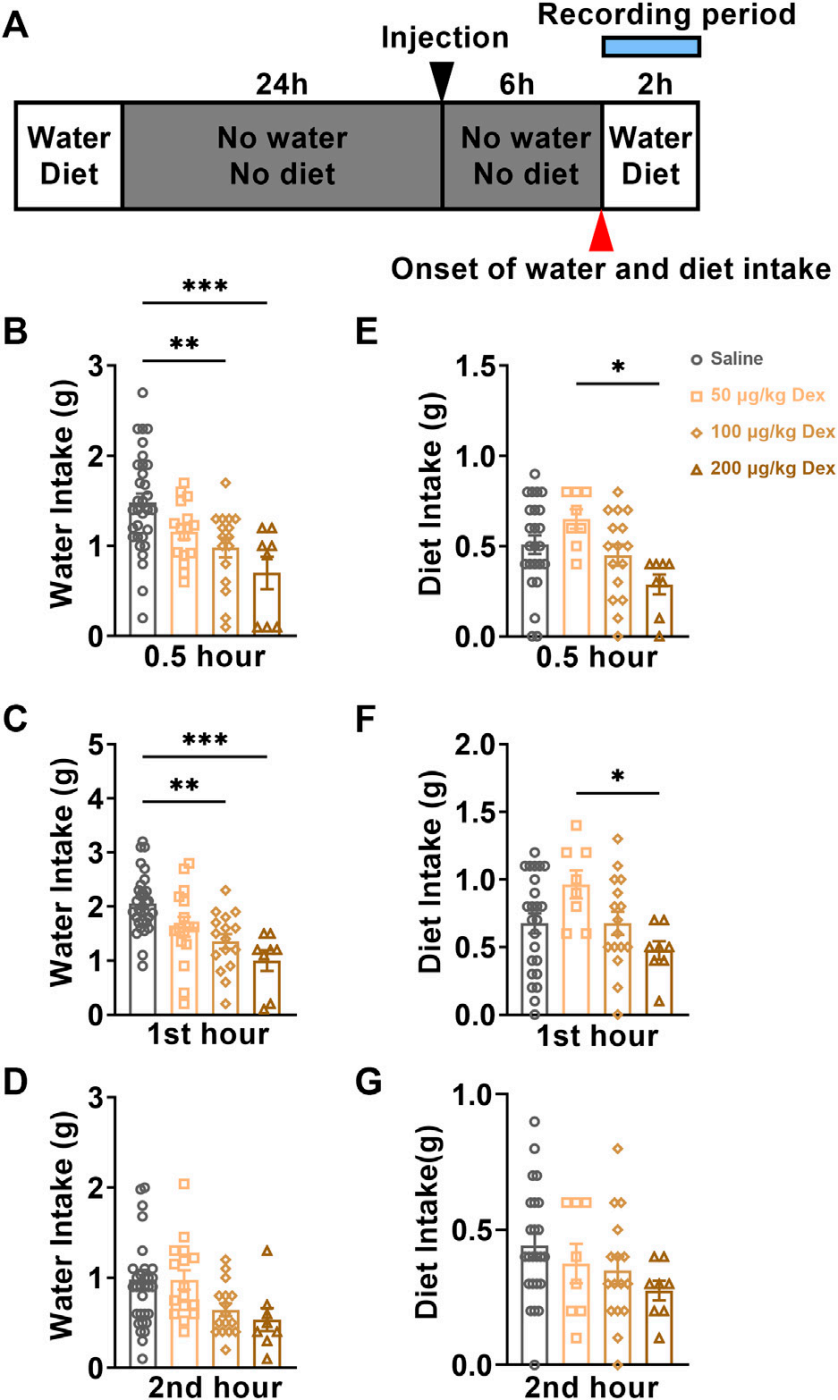
Dex dose-dependently decreases water intake. **(A)** The experimental protocol for water intake and diet monitoring after saline or Dex administration. **(B–D)** Summary of data on water consumption of each group in 30 minutes, the first hour, and the second hour after ip. Saline, ip. 50 μg/kg Dex, ip. 100 μg/kg Dex and ip. 200 μg/kg Dex (*n* = 32 in the saline group, n = 16 in the 50 μg/kg Dex and n = 16 in the 100 μg/kg Dex groups, *n* = 8 in the 200 μg/kg Dex group). **(E–G)** Summary of data on the diet of each group in 30 minutes, the first hour and the second hour after ip. Saline, ip. 50 μg/kg Dex, ip. 100 μg/kg Dex and ip. 200 μg/kg Dex. The values are presented as the mean ± SEM (*n* = 24 in the saline group, *n* = 8 in the 50 μg/kg Dex and *n* = 16 in the 100 μg/kg Dex groups, *n* = 8 in the 200 μg/kg Dex group), *p < 0.05, **p < 0.01 and ***p < 0.001 indicate significant differences among the saline and Dex groups assessed using one-way ANOVA.

The fluctuation of plasma AVP levels might affect the physiological function of water homeostasis in animals. We monitored the water and diet consumption of animals in each group for 2 h after saline or Dex administration through a customized metabolic cage (Figure 3A). During the first 0.5 h, compared to the saline group (*n* = 24), the water intake was clearly inhibited in 100 μg/kg Dex group (*n* = 16, F = 7.687, **p < 0.01) (Figure 3B) and 200 μg/kg Dex group (*n* = 8, F = 7.687, ***p < 0.001) (Figure 3B). While there is no effect on diet between Dex groups and saline group during this period, but Dex of 200 μg/kg significantly inhibited diet intake compared to the 50 μg/kg Dex group during the first 0.5 h (*n* = 8, F = 3.703, *p < 0.05) (Figure 3E). During the 1st hour, compared to the saline group (*n* = 24), the water intake was also obviously inhibited in 100 μg/kg Dex group (*n* = 16, F = 9.768, **p < 0.01) (Figure 3C) and 200 μg/kg Dex group (*n* = 8, *F* = 9.768, ***p < 0.001) (Figure 3C), while the diet intake showed no difference (*n* = 8, *F* = 2.953, *p* > 0.05) (Figure 3F). In addition, also during the 1st hour, compared to the 200 μg/kg Dex group, the diet intake was clearly more in 50 μg/kg Dex group (*n* = 8, F = 2.953, *p < 0.05) (Figure 3F) during the 1st hour. There is no difference about water intake and diet intake among these groups during the 2nd hour (Figures 3D,G). Moreover, there is no difference about water intake between saline group (*n* = 13) and 100 μg/kg Dex - 3 μg/kg AVP group (*n* = 8) during the first 0.5 h, 1st hour and 2nd hour (Supplementary Figures S1A, S1B, S1C)).

According to these results, we found that Dex of 100 μg/kg can decrease water intake without altering the diet intake. In comparison, Dex of 200 μg/kg decreased both water and diet intake, which suggested that a high concentration of Dex (200 μg/kg) inhibited drinking water may be related to the unrecovered state of anesthesia. The mice in the 100 μg/kg Dex group recovered from water intake after injection of AVP, indicating that the reduction of AVP expression played a key role in the reduction of water intake in the 100 μg/kg Dex group.

### 2.3 Dex decreases the plasma ALD level

To detect whether the Dex-induced disruption of water homeostasis would influence the function of renin-angiotensin-aldosterone pathways in modulating water intake, serum ALD, Ang II and renin concentrations were detected at the 7th hour after administration. Compared to the saline group (*n* = 8), 200 μg/kg Dex group (*n* = 10) significantly decreased serum ALD concentration (*F* = 3.756, *p < 0.05) (Figure 2C). Compared to the saline group (*n* = 8) and 50 μg/kg Dex group (*n* = 8), 200 μg/kg Dex group (*n* = 10) significantly enhanced serum Ang II concentration (*F* = 6.517, **p < 0.01) (Figure 3E). There is no difference in renin concentration among all groups (*F* = 0.4226, *p* > 0.05) (Figure 2D).

### 2.4 Dex effect electrolytes and the index of renal function in the blood

**FIGURE 4:**
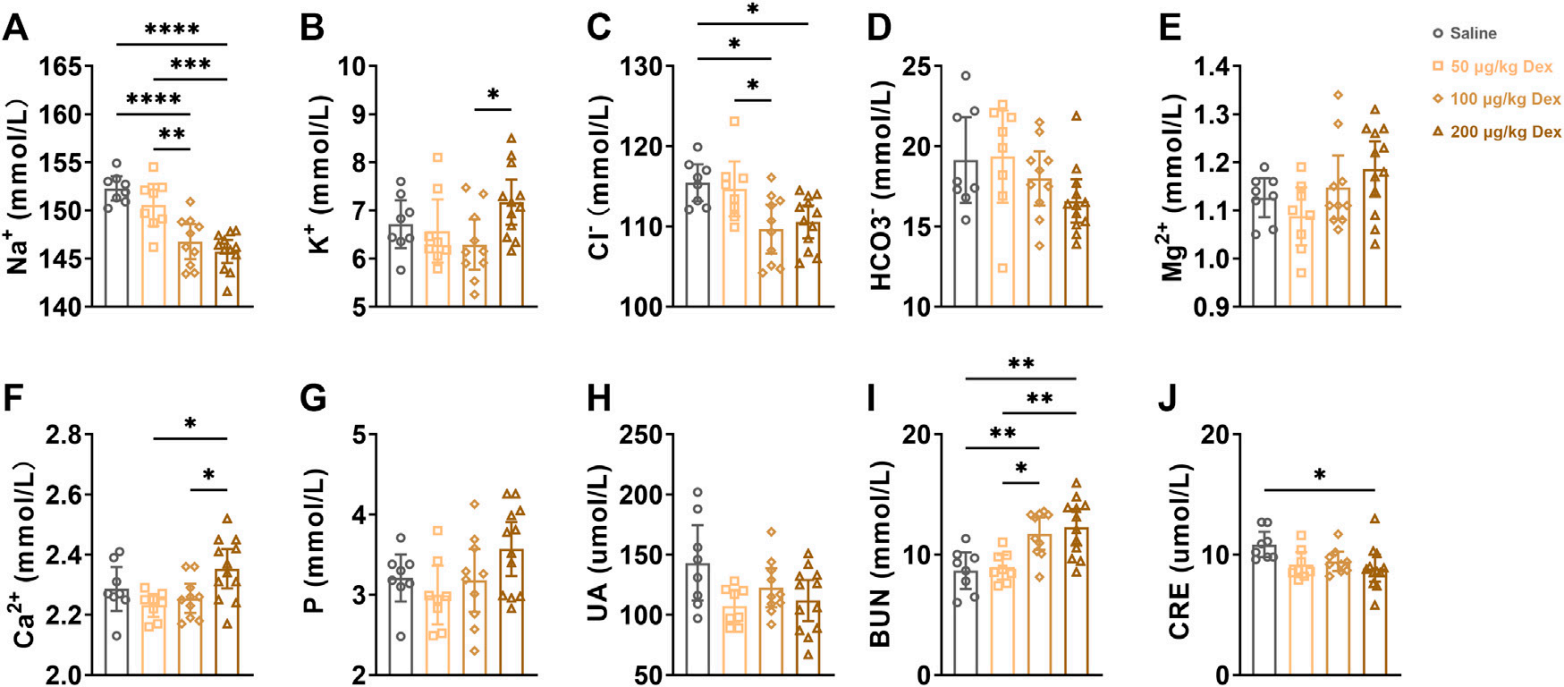
Dex effect electrolytes and index of renal function in blood(serum). **(A–J)** Summary of data on electrolytes and indices of renal function after i. p. Saline, 50 μg/kg Dex, 100 μg/kg Dex and 200 μg/kg Dex. The values are presented as the mean ± SEM (*n* = 8 in the saline and 50 μg/kg Dex groups, *n* = 10 in the 100 μg/kg Dex group, *n* = 12 in the 200 μg/kg Dex group), *p < 0.05, **p < 0.01 and ****p < 0.0001 indicate significant differences among the saline and Dex groups assessed using one-way ANOVA.

To further explore the effects of Dex on physiological function, we detected the electrolyte level in the blood as well as the index of renal function at the 7th hour after administration. Compared to the saline group or 50 μg/kg Dex group, 100 μg/kg Dex group (*n* = 10) and 200 μg/kg Dex group (*n* = 12) significantly decreased serum sodium concentration (*F* = 18.89, **p < 0.01, ***p < 0.001, ****p < 0.0001) (Figure 4A). Compared to the 100 μg/kg Dex group (*n* = 10), the 200 μg/kg Dex group (*n* = 12) significantly decreased serum potassium concentration (*F* = 2.868, *p < 0.05) (Figure 4B). Compared to the saline group, 100 μg/kg Dex group (*n* = 10) and 200 μg/kg Dex group (*n* = 12) significantly decreased serum chloride concentration (*F* = 5.793, *p < 0.05) (Figure 4C). Compared to the 50 μg/kg Dex group, 100 μg/kg Dex group (*n* = 10) significantly decreased serum chloride concentration (F = 5.793, *p < 0.05) (Figure 4C). Compared to the 50 μg/kg Dex group or 100 μg/kg Dex group, 200 μg/kg Dex group significantly increased serum calcium concentration (*F* = 4.431, *p < 0.05) (Figure 4F). Compared to the saline group or 50 μg/kg Dex group, 100 μg/kg Dex group (*n* = 10) and 200 μg/kg Dex group (*n* = 12) significantly increased serum urea nitrogen concentration (*F* = 8.795, *p < 0.05, **p < 0.01) (Figure 4I). Compared to the saline group, 200 μg/kg Dex group significantly decreased serum creatine concentration (*F* = 3.413, *p < 0.05) (Figure 4J). However, there is no difference among all groups in serum magnesium, serum phosphorus, serum bicarbonate and serum uric acid (Figures 4D,E,G,H).

### 2.5 Dex promoted urine excretion and reduced plasma osmolality

**FIGURE 5:**
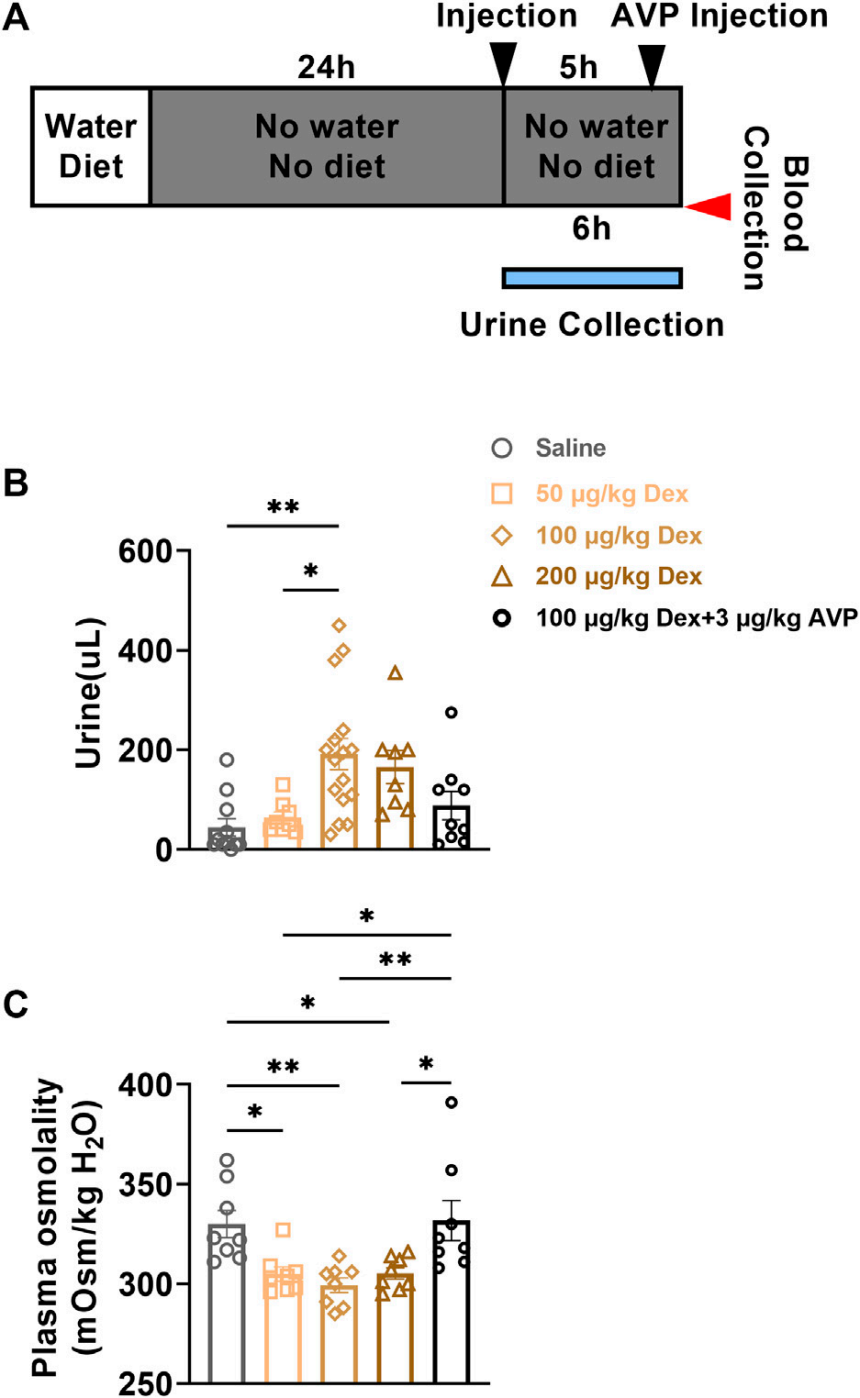
Dex increases urine output and decreases plasma osmolality. **(A)** The experimental protocol for urine and blood collection after saline, Dex or AVP administration. **(B)** Urine volume of each group within 6 h after injection of saline or Dex (*n* = 8 in the Saline, 50 μg/kg Dex and 200 μg/kg Dex group, *n* = 16 in the 100 μg/kg Dex group, *n* = 9 in the 100 μg/kg Dex-3 µg/kg AVP group). **(C)** Plasma osmolality of mice in each group 6 h after injection of saline or Dex (*n* = 8 in all groups). *p < 0.05 and **p < 0.01 indicate significant differences among the saline and Dex groups assessed using one-way ANOVA.

As Dex inhibited the expression of AVP, in order to further explore the effect of Dex on the walt-salt balance, we collected and detected the urine volume of animals in each group within 6 h after intraperitoneal injection, and then detected the plasma osmolality of animals at the seventh hour after administration (Figure 5A). Compared to saline group (*n* = 8) and 50 μg/kg Dex group (*n* = 8), the urine volume of mice in 100 μg/kg Dex group was significantly higher (*n* = 16) (*F* = 5.728, *p < 0.05, **p < 0.01) (Figure 5B) and the urine volume of mice in 200 μg/kg Dex group Showed a significantly higher trend (*n* = 8) (F = 5.728, *p* > 0.05) (Figure 5B). Compared to the 100 μg/kg Dex group, there was a significant reduced trend of the urine volume in 100 μg/kg Dex - 3 μg/kg AVP group (*n* = 9) (*F* = 5.728, *p* > 0.05) (Figure 5B). What’s more, compared to the saline group, there was no significant difference in urine volume in the 100 μg/kg Dex - 3 μg/kg AVP group. In addition, in terms of plasma osmolality, compared to the saline group (*n* = 8) and 100 μg/kg Dex - 3 μg/kg AVP group (*n* = 8), the plasma osmolality of 50 μg/kg Dex group (*n* = 8), 100 μg/kg Dex group (*n* = 8) and 200 μg/kg Dex group (*n* = 8) decreased significantly, while there was no significant difference in plasma osmolality between the saline group and 100 μg/kg Dex - 3 μg/kg AVP group (*F* = 6.494, *p < 0.05, **p < 0.01) (Figure 5C).

## 3 Discussion

Body fluid homeostasis regulates the balance of water and internal salt, AVP is the main hormone secreted from the hypothalamus to regulate body fluid homeostasis (Morimoto, 1990; Cheung and Lafayette, 2013; Christ-Crain et al., 2021). Besides, Dex is a highly selective α2-adrenergic agonist, and α2-adrenergic receptors are widely distributed in the central nervous system, especially in the hypothalamus (Unnerstall et al., 1984). Dex is indicated to inhibit the release of norepinephrine by agonizing the presynaptic membrane α2-receptor, resulting in reduced transport capacity of collecting ducts and a decrease of water permeability, especially during infusion or higher doses of Dex (Rouch et al., 1997; Mason and Lerman, 2011; Josephine et al., 2021). In this study, we found that Dex suppressed the activity of AVP neurons in the PVN, and the serum AVP concentration decreased a few hours after drug administration. In addition, ruling out the effects of food consumption, we found that Dex of 100 μg/kg suppressed water intake. Because food consumption not only influences water intake but also reflects the physical state of animals (Bankir et al., 2017). While there is no difference between the 100 μg/kg Dex group and the saline group in food intake, which shows that the inhibition of water intake induced by Dex at this concentration has nothing to do with its anesthetic effect compared to the 200 μg/kg Dex group. What’s more, 100 μg/kg Dex group mice were injected intraperitoneally with 3 μg/kg AVP 1 hour before drinking water monitoring, and water intake recovery was found, which suggested that AVP played an important role in this kind of water intake inhibition.

In addition, our urine volume monitoring results showed that Dex can significantly promote the excretion of urine, and Dex can also significantly reduce the plasma osmolality, which is consistent with the significant reduction of blood sodium content (Hoorn and Zietse, 2017). When mice injected with Dex were injected with AVP again, the phenomenon showed that urine excretion was significantly reduced, and plasma osmolality was also significantly higher than that of the Dex group at various concentrations. These above results proved that Dex increased urination and decreased plasma osmolality, which were directly related to Dex inhibiting the expression of AVP. Because AVP itself has the function of sodium and water retention. The decrease of AVP makes the excretion of water and sodium increase, urine increase, blood sodium content decrease, and plasma osmolality decrease (Sladek, 2004; Henderson and Byron, 2007; Armstrong and Johnson, 2018). Therefore, Dex increases the excretion of water and sodium on the basis of inhibiting the expression of AVP in the PVN, so as to reduce the osmolality of the body and finally inhibit thirst (Augustine et al., 2018; Gizowski and Bourque, 2018).

In mammals, ALD can promote sodium and water reabsorption and the secretion of potassium into the tubular lumen by upregulating the basolateral sodium-potassium exchange pump and the epithelial sodium channels, and the outer medullary renal potassium channels (Knepper et al., 2003; Hattangady et al., 2012; Shibata and Itoh, 2012; Briet and Schiffrin, 2013). Consistent with our results, Dex at high concentration inhibited the secretion of ALD, further decreasing serum sodium and water intake. The decrease of blood sodium was jointly affected by the decrease of AVP and ALD, and the reabsorption of sodium by renal tubules and collecting tubes was greatly reduced (Bolignano and Zoccali, 2010; Shibata and Itoh, 2012). Sodium reabsorption influences chloride reabsorption (Heerspink et al., 2016), which might be the reason why Dex significantly reduces the blood chlorine concentration. The increase in ALD concentration will also induce secondary thirst (Xanthakis and Vasan, 2013; Seccia et al., 2019). Here, the decrease in AVP and ALD concentrations was verified to decrease in water intake, but the inhibition of AVP expression may be the primary cause of Dex-induced inhibition of water intake. Moreover, the concentration of Ang II in plasma will increase with increasing Dex concentration, and it is significantly increased in the 200 μg/kg Dex group, which indicates that the body might increase the secretion of Ang II through negative feedback regulation (Franco et al., 2019; Proczka et al., 2021), Dex increases the excretion of urine and reduces the intake of water, which will lead to the reduction of blood volume (Brunkwall et al., 2020), this kind of feedback could help avoid continuous drinking inhibition (Valverde, 2021). The decrease of AVP and ALD will lead to the loss of water and salt, while the increase of AngII will stimulate the contraction of blood vessels, so as to avoid the shortage of circulating blood volume (Hussain and Awan, 2018).

Our serum electrolytes and renal function tests showed that Dex significantly increased the concentration of BUN and decreased the concentration of CRE. Dex did not significantly alter serum phosphorus, calcium, and uric acid, those electrolyte and renal function indexes we have tested all indicate that there was no substantive lesion in the renal parenchyma except serum sodium fluctuated within clinical normal ranges, for example, the normal ranges of BUN in male mice are 2.8–13.3 mmol/L (Boehm et al., 2007). In addition, the decrease in serum CRE concentration indicates an increase in the creatinine clearance rate to a certain extent, which is consistent with previous research results (Loomba et al., 2022). However, the above changes in renal function indicators suggest that Dex did not cause substantial damage to renal function.

In summary, we demonstrated that Dex could suppress water intake and inhibit the expression of AVP in the PVN, and our results also showed that AVP plays a key role in water intake inhibition. Besides, with the increase of Dex concentration, the concentration of ALD showed a decreasing trend. The high concentration of Dex significantly reduced the serum ALD level. The decrease in the ALD level will lead to secondary drinking water suppression. Our experimental results showed that Dex inhibited the expression of AVP in PVN was probably the primary factor for Dex to reduce water intake, and the weakening effect of Dex on the secretion of ALD also enhanced the inhibitory degree of water intake secondarily. Dex could promote urine excretion and decrease plasma osmolality also suggesting the truth that Dex modulates the balance of water-electrolyte metabolism by depressing the expression of AVP in PVN. In addition, Dex did not cause substantial renal damage, and the body could maintain balance through a negative feedback regulation mechanism.

## 4 Materials and methods

### 4.1 Animals

Adult male C57BL/6J mice (C57BL/6J) weighing 21–26 g were purchased from Zhejiang Wei Tong Li Hua Laboratory Animal Science and Technology Co., Ltd. All animals were housed under a T24 (12 h light:12 h dark) cycle. Food and water were obtained at room temperature (26 ± 1°C). In this study, all of the animal experimental procedures were approved by the Laboratory Animal Management and complied with the guidelines of the Committee of Shenzhen Institute of Advanced Technology, Chinese Academy of Sciences and the Laboratory Animal Management Office of Anhui Medical University. All experiments meet the guidelines and ethical requirements of the institutional nursing unit committee of Anhui Medical University.

### 4.2 Drug

Dex was dissolved in sterile saline at injection concentrations of 50 μg/kg, 100 μg/kg and 200 μg/kg. In the treatment group (*n* ≥ 4), the mice were intraperitoneally injected with Dex at 0.1 ml/10 g. In the control group (*n* ≥ 4), the mice were intraperitoneally injected with the same amount of saline solution.

### 4.3 Immunofluorescence

Three days before administration of Dex or saline solution, the mice were placed in family cages to adapt quietly and maintain a normal diet and water supply. Synchronous administration was performed at 8:00 on the day of the experiment. The protein expression time window was 90 min–120 min. Then, the mice were anesthetized with 0.04% isoflurane to reduce unnecessary pressure, perfused with 0.1 mol/L ice-cold phosphate buffer solution (PBS, pH 7.0) 50 ml through the heart, and then perfused with 4% paraformaldehyde (PFA) in 0.1 mol/L ice-cold. The brain tissue was immediately removed and fixed in 4% PFA overnight at 4°C and then transferred to 30% sucrose, and the brain tissue was kept in the solution until it sank to the bottom. Cryostat sections of whole brains were cut into 40 μm sections using a frozen slicer (Model CM1950 Leica, Germany). Brain sections were placed in 0.1 mol/L PBS at 4°C for further processing. The brain slices were washed in 0.1 mol/L PBS three times and then blocked with 5% BSA solution for 1 h. The cells were incubated with a primary goat anti-rabbit antibody against c-Fos (1:4,000 dilution, Abcam, ab190289) at 4°C overnight. After several wash steps in 0.1 mol/L PBS, the brain slices were incubated with Alexa-conjugated goat anti-rabbit secondary antibody (1:1,000 dilution in 5% BSA solution) for 2 h in darkness at room temperature. After washing several times, the brain slices were incubated with DAPI (1:2,000 dilution in PBS) and rinsed 1 time after 20 min. The brain slices were scanned under an LSM-510 confocal microscope (Zeiss). For different brain regions, we calculated the number of c-Fos positive cells in all three adjacent regions of the brain regions. Only cells stained by blue DAPI and activated by green c-Fos were selected and counted.

Brain slices containing the PVN, SCN and SON were selected and rinsed three times with 0.1 mol/L ice. Five percent goat serum was prepared with 0.5% PBST (PBS + Triton X-100) and sealed in a 37°C water bath for 30 min. The brain slices were incubated with primary goat anti-rabbit antibody against arginine vasopressin (1:1,000 dilution, Immunosotar, No. 20069) or goat anti-rabbit antibody against oxytocin (1:2,000 dilution, Immunosotar, No. 20068) overnight at 4°C. After being taken out the next day, they were rinsed several times and then added to secondary goat anti-mouse antibody (1:500 dilution in 5% BSA solution) for 2 h in darkness at room temperature. After washing several times, the brain slices were incubated with DAPI (1:2,000 dilution in PBS) and rinsed 1 time after 20 min. For these regions, AVP-positive neurons stained by blue DAPI and activated by green c-Fos were selected and counted.

### 4.4 Behavior

The water, diet and urine of the animals were detected in real time through a simple customized metabolic cage. Each metabolic cage is equipped with an independent diet and water tank, and the weight change can be accurately monitored by a precision sensor. The food and water leaked by animals in the process of eating and drinking will be automatically recovered by the device and removed in the system data at the same time to ensure the authenticity and scientificity of the results. The data were analyzed with a custom-written program running in MATLAB (MathWorks, Natick, MA). Before intraperitoneal injection, the animals in each group were deprived of water and food for 24 h and also deprived as above for 6 h after administration. The protocol is designed consistent with the preoperative preparation of patients under anesthetic in the clinic. Urine volume was measured within 6 hours after intraperitoneal injection. While the water and diet intake should be monitored from 6 h after administration, and the water and diet intake of each animal in each group should be counted in the first 30 min, the first hour and the second hour after monitoring.

### 4.5 Blood sample preparation

Before intraperitoneal injection, the animals in each group were deprived of water and food for 24 h without water and diet and also deprived as above for 6 h after administration. After 6 h of administration, the animals were anesthetized with isoflurane, If the target supernatant is serum, then the eyeballs of the mice were removed to remove the blood drop into an enzyme-free EP tube. And then incubate the EP tube containing blood in a 37 centigrade metal shower for 30 min. The blood was centrifuged by a high-speed centrifuge at 4°C and 12,000 r for 5 min, and the serum was transferred into another correspondingly labeled EP tube. In addition, if the target supernatant is plasma, the blood will drop into an enzyme-free EP tube filled with 5 µL anticoagulant EDTA-2K in advance and slightly reversed 2–3 times to mix the blood with anticoagulant. The blood was centrifuged by a high-speed centrifuge at 4°C and 1,000 r for 10 min, and the plasma was transferred into another correspondingly labeled EP tube.

### 4.6 Blood analysis

The mice were sacrificed by decapitation, and plasma was collected using EDTA-2K after centrifugation. According to the manufacturer’s instructions, serum AVP was measured by ELISA. Serum ALD, Ang II and renin levels were measured by the magnetic particle chemiluminescence method combined with a chemiluminescence detector (AutoLumo A2000). Magnetic particles were coated with a second antibody, ALD antibody was used to prepare the antibody solution, and horseradish peroxidase (HRP)-labeled ALD antigen was used to prepare the enzyme conjugate to prepare the second antibody-enzyme labeled antigen complex. The serum ALD concentration was detected by the fluorescence intensity being inversely proportional to the ALD concentration. Magnetic particles were coated with Ang II, Ang II antigen was labeled with biotin, and avidin was labeled with HRP. The HRP-antigen-antibody complex was formed by an immune reaction, and the concentration of serum Ang II was detected by its fluorescence intensity, which was inversely proportional to the concentration of Ang II. The magnetic particles were coated with renin antibody, and HRP was used to label the renin antibody to prepare the enzyme conjugate. The solid-phase antibody-antigen-enzyme conjugate was formed by an immune reaction, and the serum renin concentration was detected by using the fluorescence intensity of the conjugate proportional to the renin concentration. Serum electrolyte and renal function indices were measured by a Vitros 250 chemical analyzer. Plasma osmolality was determined from the method of freezing point depression using the semi-automated Advanced Micro-osmometer (Advanced Instruments Inc., Norwood, MA, United States).

### 4.7 Statistical analysis

All statistical analyses were performed using SPSS 21.0 software (IBM, Almonk, New York, United States) and GraphPad Prism 8.0 software (GraphPad, San Diego, CA, United States). The comparisons of measured data and the number of “active” cells in different groups are presented as the mean ± SEM. Student’s *t* test was used to analyze the comparison among different groups. One-way ANOVA was used to analyze the same drug in groups with different doses (the number of groups is more than 2). For all tests, a value of *p* < 0.05 was considered statistically significant.

## Data Availability

The original contributions presented in the study are included in the article/Supplementary Material, further inquiries can be directed to the corresponding authors.
