# Supplementary figures and images for: Dex modulates the balance of water-electrolyte metabolism by depressing the expression of AVP in PVN
Source: Front Pharmacol. 2022 Aug 23;13:919032. doi: 10.3389/fphar.2022.919032 (PMC9445239; doi:10.3389/fphar.2022.919032)

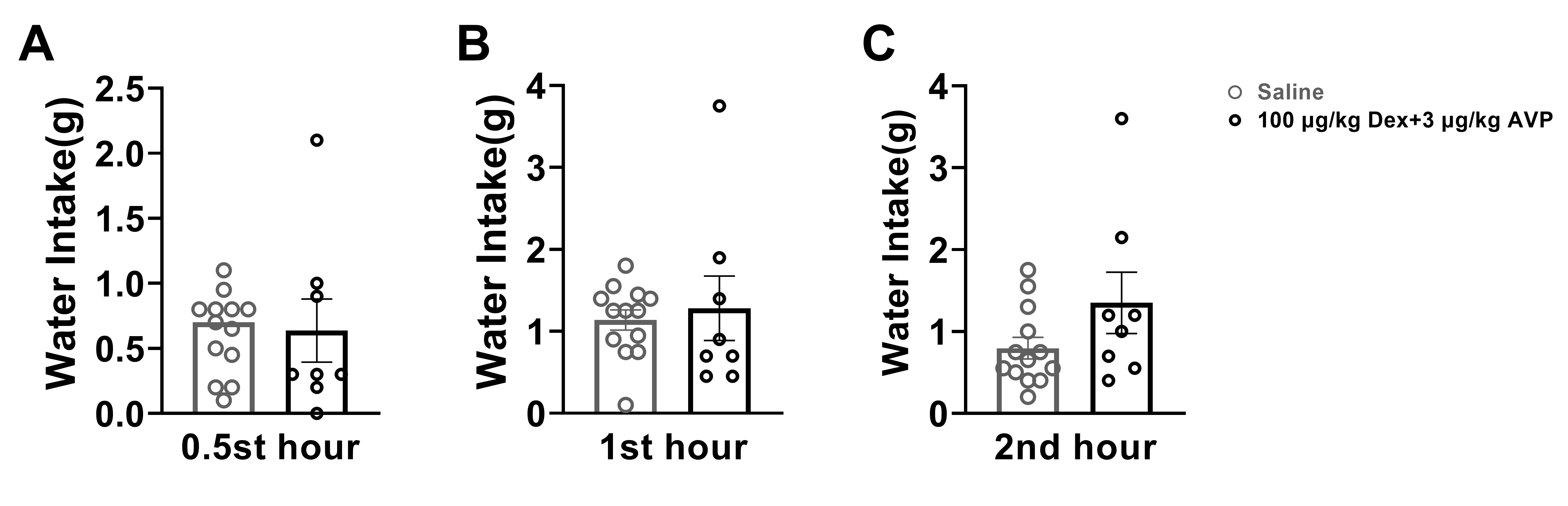

Supplement: Supplementary file 1 [file Image1.tif]
